# Research on Distance Transform and Neural Network Lidar Information Sampling Classification-Based Semantic Segmentation of 2D Indoor Room Maps

**DOI:** 10.3390/s21041365

**Published:** 2021-02-15

**Authors:** Tao Zheng, Zhizhao Duan, Jin Wang, Guodong Lu, Shengjie Li, Zhiyong Yu

**Affiliations:** State Key Laboratory of Fluid Power and Mechatronic Systems, Zhejiang University, Hangzhou 310027, China; zhengtao2955@zju.edu.cn (T.Z.); duanai@zju.edu.cn (Z.D.); lugd@zju.edu.cn (G.L.); 11825057@zju.edu.cn (S.L.); k1062@zju.edu.cn (Z.Y.)

**Keywords:** semantic segmentation, distance transform watershed, neural network, classification, lidar information, mobile robot, Raspberry Pi, 2D lidar room map

## Abstract

Semantic segmentation of room maps is an essential issue in mobile robots’ execution of tasks. In this work, a new approach to obtain the semantic labels of 2D lidar room maps by combining distance transform watershed-based pre-segmentation and a skillfully designed neural network lidar information sampling classification is proposed. In order to label the room maps with high efficiency, high precision and high speed, we have designed a low-power and high-performance method, which can be deployed on low computing power Raspberry Pi devices. In the training stage, a lidar is simulated to collect the lidar detection line maps of each point in the manually labelled map, and then we use these line maps and the corresponding labels to train the designed neural network. In the testing stage, the new map is first pre-segmented into simple cells with the distance transformation watershed method, then we classify the lidar detection line maps with the trained neural network. The optimized areas of sparse sampling points are proposed by using the result of distance transform generated in the pre-segmentation process to prevent the sampling points selected in the boundary regions from influencing the results of semantic labeling. A prototype mobile robot was developed to verify the proposed method, the feasibility, validity, robustness and high efficiency were verified by a series of tests. The proposed method achieved higher scores in its recall, precision. Specifically, the mean recall is 0.965, and mean precision is 0.943.

## 1. Introduction

Nowadays, people are increasingly interested in the field of mobile robots. This is because mobile robots can help people accomplish more and more tasks. For instance, mobile robots can work in tasks such as elderly care, guidance, office and domestic assistance, inspections and many more. Mobile robots usually work in indoor environments designed for humans, with offices and houses being some of the most typical examples [1]. Dividing the complex navigation maps or floor plans into simple cells is playing a more and more important role in many tasks executed by mobile robots because robots need to understand the environment so that they can complete their missions smoothly. For example, with the help of semantic maps, robot can obtain navigation trajectories only requiring a small amount of computation [2].

Besides, one of the other main uses of indoor room semantic segmentation is automatized professional cleaning [3]. In this task, a sweeping robot need to clean the floor in indoor rooms. After dividing a room into simple semantic cells the sweeping robot can perform cleaning tasks in each unit more autonomously and intelligently. The reasonable segmentation of complex room maps into simple units can make the robots plan the cleaning path faster, and make the whole cleaning task perform better.

Various methods of 2D indoor room map segmentation have been proposed in recent years. The segmentation of individual room units from floor plans can based on the semantic mapping [4] or places classification [5]. Morphological segmentation is described in [6,7,8,9,10]. It initializes the walls in the map to expand and break down the interconnected area until all areas are blocked into different small cells. Major highlights of this method are the high computing speed and algorithmic simplicity. However, it achieves poor performance when the shape of the room is not very regular. The distance transform watershed-based segmentation method [11,12,13,14] divides the room by applying a distance transform to find the room centers at optimal threshold, then segmenting with a wavefront propagation. Besides, the lidar point clouds information and laser scanning data are also widely used to classify and segment the maps [15,16,17], but because of its high computational complexity, it’s still a big challenge for low memory consumption devices like the Raspberry Pi. Another approach is a Voronoi graph-based segmentation [2,18,19,20,21,22,23,24], which extracts the critical points and lines to divide the Voronoi cells from the Voronoi graph generalized by the original rooms, then segments the room after merging Voronoi cells. It is the most popular approach to segment floor plans and performs well.

However, none of these methods can obtain the semantic labels of the room maps. The segmentation of grid maps into semantically meaningful areas is an important task for many mobile robot applications. It usually enables the robot to understand the current environment and make decisions by obtaining the semantic information of the room maps. For instance, if the sweeping robot can obtain the semantic information of the navigation map, it can plan the cleaning order of each room based on the location of the doorways, and achieve higher sweeping performance in a shorter time. It depends largely on how can robot understand the semantic information of room whether the interaction between humans and robots can be proceed efficiently [6,25].

In the particular case of indoor environments, we can find typical semantic divisions of 2D lidar maps such as corridors, rooms, or doorways. Taking these factors into account, a feature-based segmentation [1,4,26,27,28] was reported, which simulated a laser scanner measurement within the navigation maps, then segmented the maps by AdaBoost classification. This method can obtain the semantic information of each point on the map by classifying each point. However, it is time-consuming to sample every point on the maps and run the classification method, and these kinds of classification methods are difficult to run on the sweeping robot with low computing power. What’s more, the robustness of the algorithm is not good. Besides, Kleiner, A. [14] proposed a method can get the rooms and doorways semantic labels of the map, but they are labeled by a human via a cloud service and phone/tablet APP.

In this work, in order to get a better, faster and more effective semantic segmentation method we combine distance transform watershed-based pre-segmentation and a skillfully designed fast neural network sampling classification method to design a low-power and high-performance method to label 2D lidar room maps. In the training stage, we simulate a lidar to collect the lidar detection line maps of each point in the manually labelled map, and then use these line maps and corresponding labels to train the designed neural network. In the testing stage, we first pre-segment the new map into various simple cells with the distance transform watershed method, then by using the result of distance transform generated in the pre-segmentation process the optimized areas of sampling points are selected. Then we classify the lidar detection line maps sampled from optimized areas with the trained neural network and the “winner-take-all” principle. Compared with the distance-transform based method, Voronoi and morphological segmentation method, our method can not only obtain the semantic information of maps, but also still run efficiently. Compared with the widely used ResNet-18 neural network, our method performs better and runs faster.

The rest of this paper is arranged as follows: Section 2 describes the related works about our method. The proposed architecture of our method is discussed in the Section 3. The main experimental results and analysis are introduced in Section 4. Section 5 concludes the paper.

## 2. Related Works

### 2.1. Semantic Labels

Understanding the semantic information of rooms is an important task for many mobile robot applications. An office sweeping robot can visit all rooms in an optimal order by utilizing a map segmentation. Most of the relative approaches divides the indoor rooms into three categories: “rooms”, “doorways” and “corridors” [1] because these are the three most representative semantic labels on a 2D map. We also segment the rooms into the three categories in our approach.

### 2.2. Deep Learning for Classification

With the rapid development of deep learning, the accuracy of image classification has been significantly improved. Many excellent network architectures such as VGGNet [29], GoogleNet [30,31], ResNet-18 [32] and MobileNet [33] have been proposed to solve the problem of image classification. In particular, the classification performance of ResNet-18 on ImageNet datasets has exceeded the performance of human beings. However, all these networks rely on the powerful computing power of GPUs and large datasets, which makes it difficult to deploy them on low-power edge devices. Especially for the sweeping robot, which requires fast and efficient performance but its computing power is very poor. In order to apply the powerful classification ability of deep learning, we propose a lightweight classification network of lidar line maps that can be deployed on Raspberry Pi 3B+ devices. The network classifies doorways, rooms and corridors on maps by learning the features of virtual lidar data emitted from different points on maps. Moreover, the lightweight model architecture and sampling classification method effectively ensure the high performance of the algorithm.

### 2.3. The Distance Transform Watershed Based Pre-Segmentation

A classic way of separating touching objects in binary images makes use of the distance transform and the watershed method. The idea is to create a border as far as possible from the center of the overlapping objects. This strategy is called distance transform watershed. It consists of calculating the distance transform of the binary image, inverting it (so the darkest parts of the image are the centers of the objects) and then applying watershed on it using the original image as mask.

**Figure 1:**
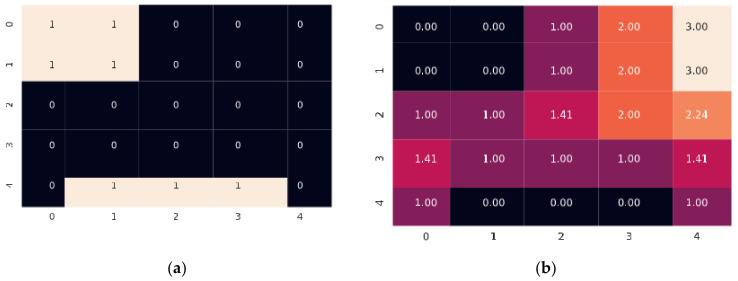
(**a**) Binary map matrix; (**b**) Distance transform matrix.

In order to improve the performance of the architecture, we use the distance transform watershed to pre-segment the maps. A distance transform represents the distance of each accessible (white) pixel to the closest border pixel (black). In below, Figure 1a shows a binary map matrix, and in Figure 1b shows the corresponding distance transform matrix.

**Figure 2:**
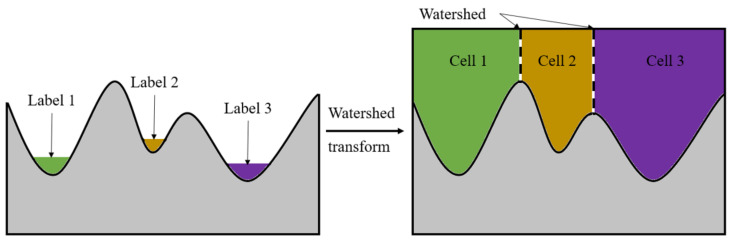
The segmentation process of the watershed algorithm.

After the corresponding distance transform matrix is obtained, the pre-segment can be used with the watershed method by setting an appropriate threshold. The watershed segmentation method is a kind of mathematical morphology segmentation method based on topology theory, the basic idea is to put the image as the topology of landform on geodesy, each pixel grayscale value in the image indicates that point elevation, each local minimum values and effect area known as the reception basin, and set the boundary of the basin form a watershed. Figure 2 shows the segmentation process of the watershed algorithm.

## 3. Proposed Method

In this work, we propose a novel approach to get the semantic labels of room maps which consists of two components. In the training stage, the original room map is binarized and manually labelled into three categories (the labelled map): room, corridor and doorway. Then, a simulated lidar goes through all white areas of the map. The lidar line data of each point in the maps and corresponding labels are collected for semantic classification. In order to complete the classification tasks efficiently, a light-weight network named as LCNet is designed and trained with the map data, which is inspired by LeNet [34] and can run in the Raspberry Pi 3B+.

**Figure 3:**
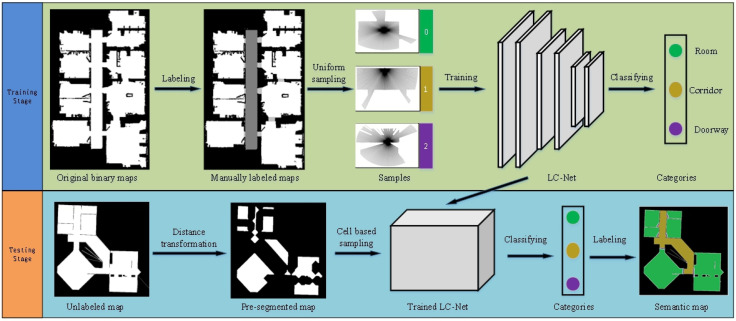
The overall framework of the proposed method.

In the test stage, the unlabelled binary map is pre-segmented into many closed simple areas firstly. Then we sample the lidar line data uniformly in each distance transformed pre-segmented area. We use the data sampled in the distance transformed pre-segmented area, which gives a greater distance between classes in rooms, corridors and doorways. Next, the optimized areas based sampling data are inputted into the trained LCNet for classification. Finally, the semantic information in each cell are obtained according to the proposed “winner-take-all” principle. The overall framework of our method is illustrated in Figure 3.

### 3.1. Laser Data from Simulated Lidar

**Figure 4:**
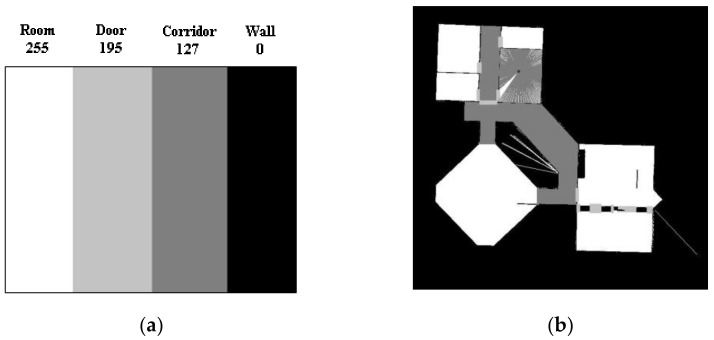
(**a**) shows the four labels and its grayscale values: 255 for rooms, 195 for doorways, 127 for corridors, and 0 for walls, (**b**) shows the simulated laser scanner measurement within the manually labelled map.

The performance of a neural network is greatly affected by the amount of data, but there are few 2D room maps. It is very difficult to train a neural network to segment these maps based on just a few labelled maps, so we turn it into a classification problem inspired by Mozos’ research [4]. In the process of laser SLAM building of 2D maps, the 2D map is built by laser scanning. In the same way, we can use a simulated robot to extract the laser map information of each point in the maps. The information of each point can provide a large training data set. By classifying the image information of each position, we can realize the semantic labeling of 2D area. The original 2D map built by a 2D lidar is manually labelled into four kinds of regions with four different grayscale values. The details are shown in Figure 4.

**Figure 5:**
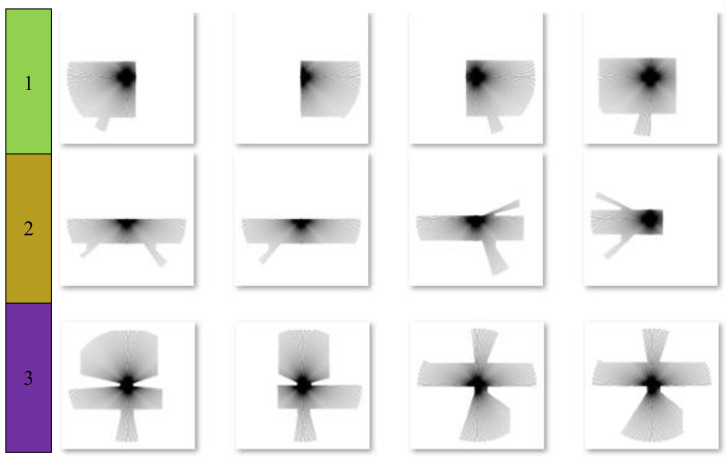
The laser map information collected from different kinds of areas. The room is labelled as 1, the corridor is labelled as 2, and the doorway is labelled as 3.

Our simulated robot is equipped with a 360° field of view laser sensor. Each laser observation consists of 360 beams. With the robot traveling to all the areas where the grayscale values of labels are bigger than zero, as shown in Figure 4b, the laser map information and labels in every point are uniform sampled as the training data. As shown in Figure 5, the laser map information collected from different kinds of areas reflect various kinds of appearance information. The beams of corridors are usually long and narrow, while those of rooms are wide and round. With access to ample data, we can train a powerful classifier based on a neural network.

### 3.2. The Optimized LCNet Network

Nowadays, deep neural networks have become one of the most powerful feature extraction methods. The most widely used is ResNet-18 because this work are very deep compared with previous networks. A deeper feature extraction network can learn more advanced features and can classify better, as has been shown by researchers in recent years. However, substantial computing power is required to implement an efficient network such as ResNet-18, which is a big challenge for low memory consumption devices like Raspberry Pi. In order to reduce the parameters and computing power required by the modelso it can be deployed to a low computing power device, a lightweight network architecture is designed based on ResNet-18 named as LCNet.

In our training stage, the first difference between original ResNet-18 and our LCNet is that we delete the 7 × 7 conv in the first layer, because the features in the low-level layer learning are not enough, while this layer requires a substantial computing power. What’s more, we use a smaller input of 48 × 48 instead of the 112 × 112 one in the original model.

Secondly, as shown in Table 1, we replace the original ResBlock with LCBlock. The LCBlock is a depthwise style block with an Octconv layer. Deepthwise convolution has been widely used since it was first proposed in [30]. As an efficient convolution method to reduce the number of parameters and ensure the accuracy, we apply this conv layer instead of a normal conv layer. As a substitute of common convolution, Octconv greatly reduces the memory and computing resources needed by reducing the resolution of low frequency images [32], so we use the Octconv to extract the features.

**Figure 6:**
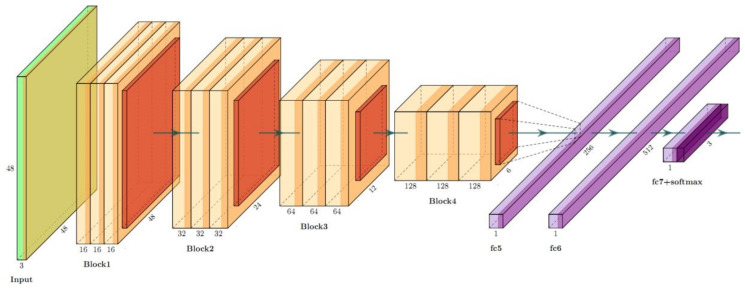
The framework of proposed LCNet Block.

By combining the blocks, we build the LCNet. The LCNet contains of four groups of blocks, as shown in Table 2, and Figure 6 shows the layers and the framework of LCNet of our proposed training stage.

### 3.3. Classification Based on Pre-Segmentation and Optimized Sampling Areas

#### 3.3.1. Pre-Segmentation

In the training stage, by scanning and classifying each point with the proposed LCNet, we can reconstruct a semantic segmentation map (SSM) to know the labels of each point. There are thousands or much more pixels in a map, which means that there are sufficient data for training the network. However, in the actual testing stage, it would take too much time to scan and classify each point. Moreover, limited by the performance of the lightweight neural network model and low computing power devices, the recognition rate is not very high, which will lead to several different classification results for the same area, that is, the recall rate of recognition results in the same area is not good enough.

Therefore, in the testing stage, it is obviously not desirable to use the point-by-point recognition method alone for semantic segmentation.

**Figure 7:**
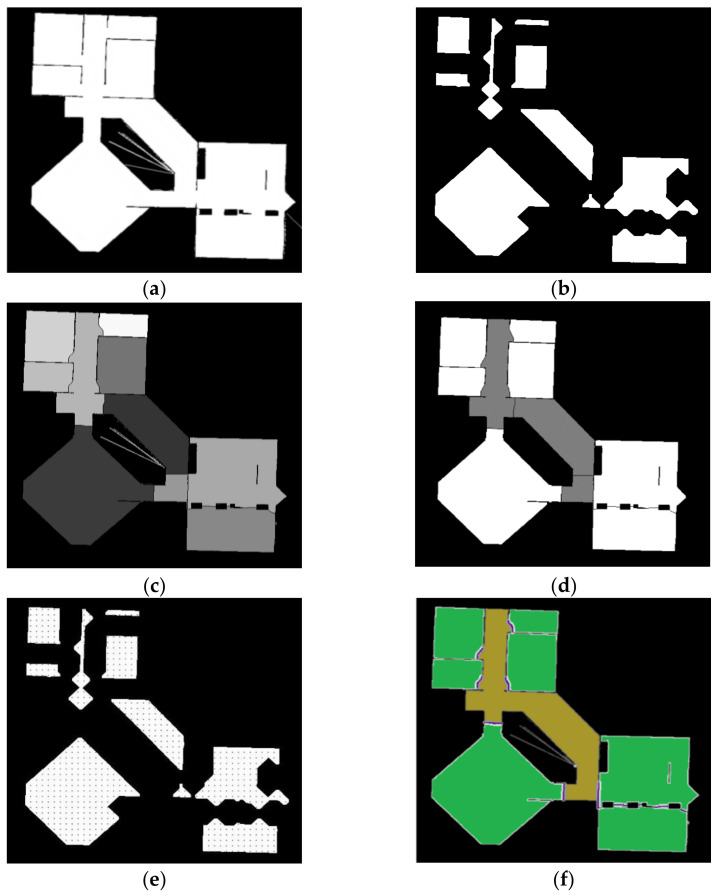
(**a**) is the binary pre-processed map (PPM), (**b**) is the result of distance transformation, (**c**) is the pre-segmented map (PSM) processed by distance transformation and watershed algorithm, (**d**) is the map with room and corridors labels from automatic classification, and (**e**) is the sampling diagram in the optimized sampling areas, (**f**) is the result of Semantic Segmented map.

In this work, by combining with distance transform and watershed pre-segmentation, the speed and regional consistency of semantic segmentation can be greatly improved. Specifically, we use distance transformation and a watershed algorithm to pre-segment the map into different cells at first, as shown in Figure 7b,c.

Then a specific number of points are selected in the pre-segment area, we sample the points every 0.05 m in the four directions of up, down, left and right in each area, and only these sparse sampling points can be scanned and classified, as shown in Figure 7e. Then the classification results of the sampling points are counted.

According to the “winner-take-all” principle, the label with the highest proportion in each area is adopted as the unified label of this area, thus we get the result shown in Figure 7d, in Algorithm 1 we show that how the “winner-take-all” principle is implemented.
**Algorithm 1** Labeling room areas and corridors areas with “winner-take-all” principle**Input:** Pre-processed map(PPM), Pre-segmented map(PSM), classification results of sampling points; **Output:** The map with room labels and corridors labels; 1: The classification results of sampling points in each area were counted. m is the number of sampling points identified as rooms in each area, and n is the number of sampling points identified as corridors; 2: **for** each area of the binarized map **do** 3:   **if**
*m* >= *n*
**then** 4:     Classify this area as room; 5:   **else** 6:     Classify this area as corridors; 7:   **end if** 8: **end for**

#### 3.3.2. Optimized Sampling Areas and the Extraction of Doorway Labels

What should not be ignored is that since SSMs of the points distributed at the junctions of rooms and corridors have very similar features to each other, the recognition accuracy of these points in junction areas will be reduced significantly.

In order to prevent the sampling points selected in the boundary area from influencing the results of semantic labeling, the optional range of sampling points should be narrowed to exclude the boundary area, for this purpose, in this work the optimized area of sampling points is proposed by using the result of distance transformation generated in the pre-segmentation process.

After distance transformation and binarization, each cell is reduced to a smaller area around its geometric center, as shown in Figure 7b, and in this work these areas are adopted as optimized areas of sampling points meeting our requirements, as shown in Figure 7e. Only these sparse sampling points selected from the optimized areas can be scanned and classified, which can significantly improve the recognition accuracy and the calculation speed.

Besides, from Figure 7d we can find that only rooms and corridors can be distinguished during the pre-segmentation process, but the area of the doorway was not distinguished. This is because we only do the sampling classification in the optimized sampling area, and do not sample at the junctions areas of rooms and corridors. So the next is the extraction of doorway labels.

In fact, we can find that pixels with the label of doorway are distributed along the dividing line between the different areas, however, watershed algorithm is prone to over-segmentation, so we cannot simply label all dividing lines as doorways, we need to classify the points on the dividing lines, so the first step is to determine all the dividing lines, that is, to find out all the points on the dividing line. Specifically, comparing the pre-processed map Figure 7a with the map shown in Figure 7d point by point, and find out all the pixels on the dividing line.

The next step is to determine which dividing line the pixel belongs to. According to the information of Figure 7d, the grayscale value of areas on both sides of each dividing line can be obtained, since the grayscale value of each area in the pre-segmented map is unique, it can be used to determine which dividing line the pixel belongs to.

The third step is to determine the semantic label of the dividing line. In this work, we automatically mark the dividing line between different semantic areas as doorway without classification, which can speed up the calculation significantly. While the points on the dividing line between the same semantic areas are classified by the trained network. Then according to the “winner-take-all” principle proposed above, if the proportion exceeds the set value, we mark the dividing line as a doorway. We describe the above steps in detail in Algorithm 2.
**Algorithm 2** Labeling doorway areas**Input:** Pre-segmented map (PSM), pre-processed map (PPM), classification results of sampling points, the map with room labels and corridors labels; **Output:** Result of semantic segmentation; 1: Extracting the size of pre-processed map, define *rows* as number of rows and *cols* as number of columns, define two-dimensional vector *dl_type* to store grayscale on both sides of the dividing line, define vector *dl_n* to store the number of pixels of each dividing line, define vector *dl_d* to store the number of pixels with “doorway” label of each dividing line; 2: **for**
*x* in [0, *cols* - 1] **do** 3:   **for**
*y* in [0, *rows* - 1] **do** 4:     **if** PPM(*x*, *y*) != 0 && PSM(*x*, *y*) == 0 **then** 5:       **if** size(*dl_type*) == 0 **then** 6:         Push {*g1*, *g2*}into *dl_type*, where *g1* and *g2* are gray values of the both sides of the pixel (*x*, *y*) respectively; 7:         Push [33] into *dl_n*, push [33] into *dl_d*; 8:         **if** pixel (x, y) is classified as “doorway” **then** 9:           *dl_d*[0]++; 10:         **end if** 11:       **else** 12:         **if** {*g1*, *g2*} exists in *dl_type*
**then** 13:           *dl_n*[*i*]++, where *i* is the corresponding subscript when *dl_type*[i] == {g1, g2}; 14:           **if** pixel (x, y) is classified as “doorway” **then** 15:             *dl_d*[*i*]++; 16:           **end if** 11:         **else** 12:           Push {*g1*, *g2*} into *dl_type*, push [33] into *dl_n*, push [33] into *dl_d*; 13:           **if** pixel (x, y) is classified as “doorway” **then** 14:             *dl_d*[size(*dl_d*) - 1]++; 15:           **end if** 16:         **end if** 17:       **end if** 18:     **end if** 19:   **end for** 20: **end for** 21: Define the threshold *thr_d*; 22: **for**
*j* in [0, size(*dl_type*) - 1] **do** 23:   **if**
*dl_d*[*j*] / *dl_n*[*j*] >= *thr_d*
**then** 24:     Mark the *j*th dividing line as doorway; 25:   **else** 26:     Erase the *j*th dividing line; 27:   **end if** 28: **end for**

## 4. Experiments and Analysis

**Figure 8:**
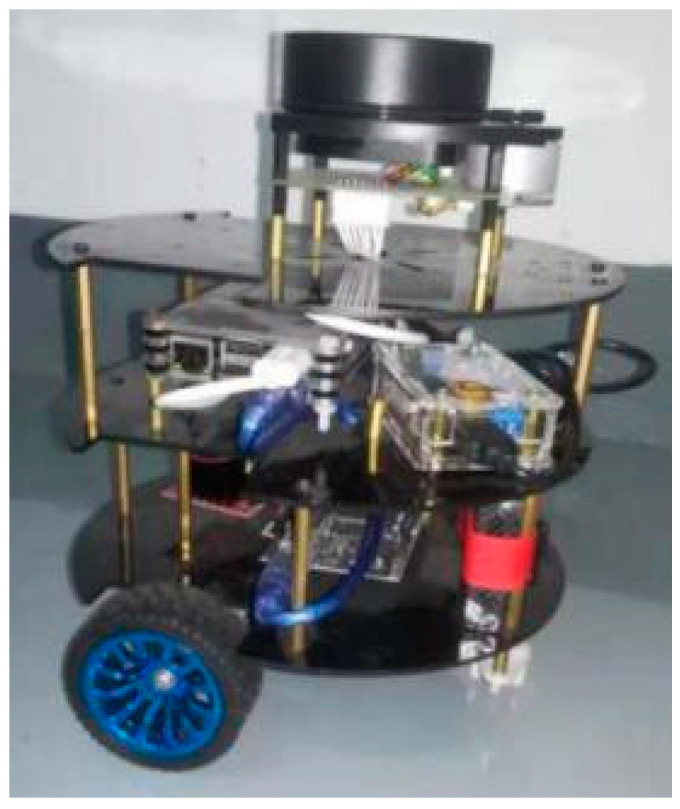
The designed mobile robot with 2D lidar.

The proposed method has been tested on real robots as well as in simulation. The robot used to carry out the experiments is a mobile robot equipped with a 2D laser (RPLIDAR-A1, SLAMTEC, Shanghai, China), which can perform 360° scans within 12-m range and generate 8000 pulses per second. This system supports programming with Raspberry PI and the Arduino toolkit, as shown in Figure 8. With its compact structure, the robot can move through the room flexibly, and with the adoption of Gmapping package in a ROS framework, it can complete the SLAM task reliably.

The goal of our experiments is to demonstrate that our method is a robust 2D segment framework. Firstly, we compare the accuracy and the speed of LCNet and ResNet-18, which proves that our proposed LCNet can learn the laser data well and performs well. Secondly, we verify the performance of the segmentation effect based on pre-segmentation and optimized sampling areas by applying the proposed method to different 2D maps. Then we test the semantic segmentation performance of our algorithm and compare it with current mainstream methods.

### 4.1. Results of the LCNet and the ResNet-18

**Figure 9:**
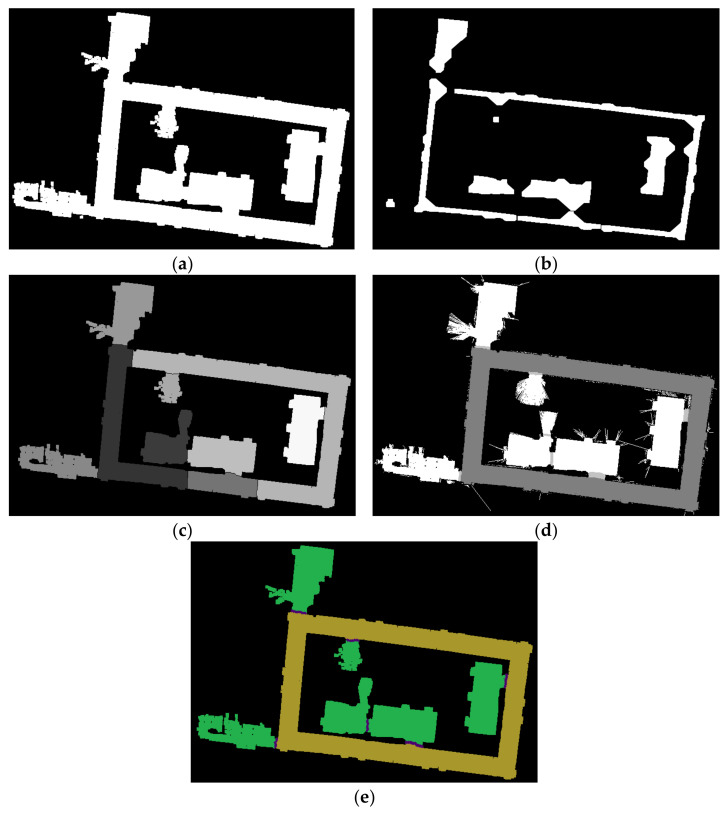
(**a**) is the binary pre-processed map (PPM), (**b**) is the result of distance transformation, (**c**) is the pre-segmented map (PSM)processed by distance transformation and watershed algorithm, (**d**) is the map with room and corridor labels, and (**e**) is the result of Semantic Segmented map.

The first experiment was tested using real data from a lab environment in our labs to compare the performance of ResNet-18 and proposed LCNet, the lab map is shown in Figure 9a. We first get our lab’s 2D map with the designed mobile robot by using Gmapping method. Then we label the 2D lab map with four different grayscale values shown in Figure 4a based on the map’s real segmented class. A simulated lidar goes through all the white areas. The 80% lidar line data of the map and the corresponding labels are collected as training data. The remaining 20% is used for testing data. Figure 9 shows the process of the experiment.

By sampling the laser data of each pixel, we collect all 40,950 laser line data in the labelled 2D lab map, 80% of which is collected for training data. The remaining 20% is used for testing data. We train the LCNet and the ResNet-18 model in GPUs and test them in a PC and the Raspberry Pi 3B+ with the same data. ResNet-18 is only tested on the PC because the Raspberry Pi’s computing power is not yet sufficient for ResNet-18 testing. In the training stage, we using the Adam optimizer, learning rate of 0.01, batch-size of 64, and iterated for 20 epochs on an NVIDIA 1070Ti 8G GPU.

**Figure 10:**
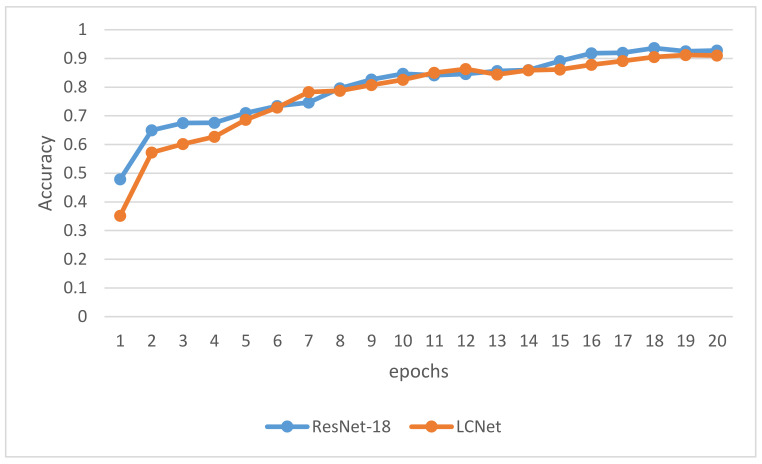
The accuracy results of the ResNet-18 and LCNet in the iterative training.

As shown in Figure 10, after 16 epochs of iterative training, ResNet-18 has reached a state of convergence, and its classification accuracy on the test set has reached a stable state. After 18 epochs of iterative training, ResNet-18 has achieved the highest classification accuracy of 93.6% in the 18th epoch. The LCNet converges after training for 18 epochs, and achieves the highest classification accuracy of 91.2% at the 18th epoch. After convergence, the classification accuracy of LCNet is 1.4% lower than that of ResNet.

**Figure 11:**
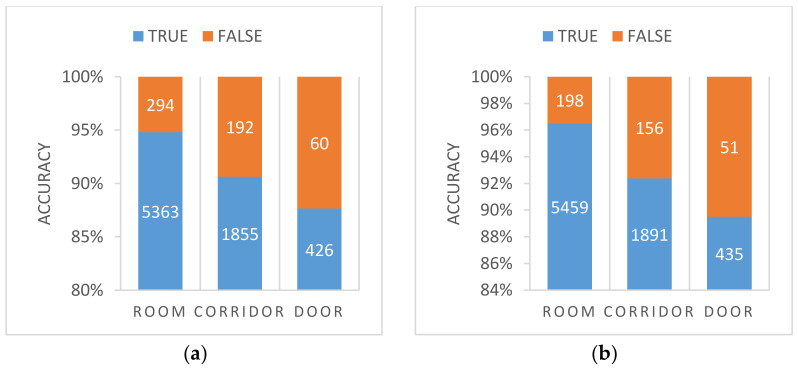
(**a**) is the accuracy of the LCNet, (**b**) is the accuracy of the ResNet.

Then we compared the 18th epoch model of LCNet and the 16th epoch model of ResNet, the classification results of the two models on each category of the test set is analyzed as shown in Figure 11. “TRUE” indicates the number of samples with correct predictions and “FALSE” indicates the number of samples with wrong predictions.

It can be found that the accuracy rates for rooms, corridors, and doorways on LCNet networks were 94.8%, 90.6%, and 87.6%, respectively, while for ResNet-18 they were 96.5%, 92.4%, and 89.5%, respectively, i.e., slightly higher than with LCNet, but in terms of running time, we compare the test time of LCNet and ResNet-18, as shown in the following Table 3.

According to Table 3, the speed of LCNet is 3.37 times faster than ResNet-18 on a PC, so the speed is significantly improved. Moreover, the size of the model is significantly reduced, and it can be extended to run on a Raspberry Pi device, while ResNet-18 cannot be deployed on Raspberry Pi devices. However, because the test images are sampled pixel by pixel and then converted into pictures, 8190 points need to be classified, so the calculation on Raspberry Pi are time-consuming. By analyzing the characteristics of the images of adjacent sampling points, we find that their features are very similar, so we can reduce the number of classification points and improve the running speed by the proposed sparse sampling above.

### 4.2. Results of the Proposed Classification Method

**Figure 12:**
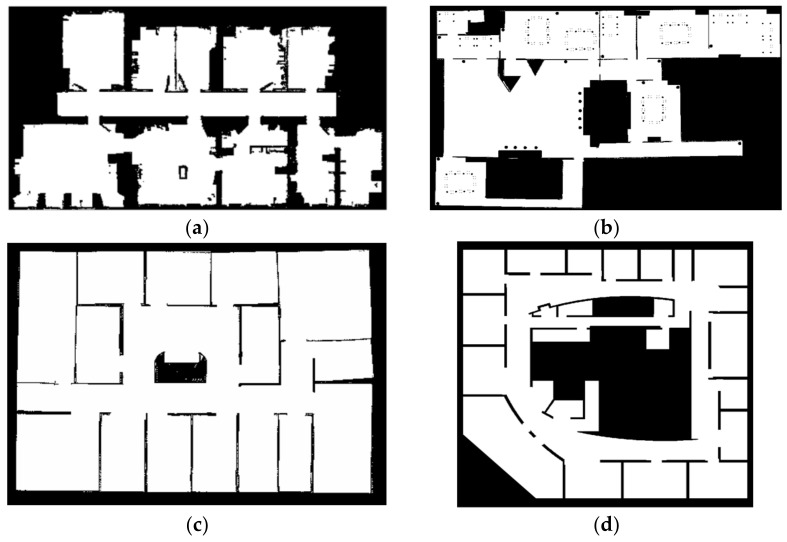
(**a**) freiburg_building52 map, 6986 points are sampled. (**b**) lab_d map, 12,630 points are sampled. (**c**) is lab_c map, 9852 points are sampled. (**d**) is lab_intel map, 15,368 points are sampled.

As mentioned above, in this paper we have proposed some optimizations using the distance transform watershed method to pre-segment the map first, and then evenly collecting the sample points in each optimized sampling area and inputting them into the proposed LCNet for classification, and then according to the “winner take all” rule, the final semantic labels of the classification results are determined. We used four maps as the training data set for the proposed LCNet, as shown in Figure 12, which are publicly available [3].

**Figure 13:**
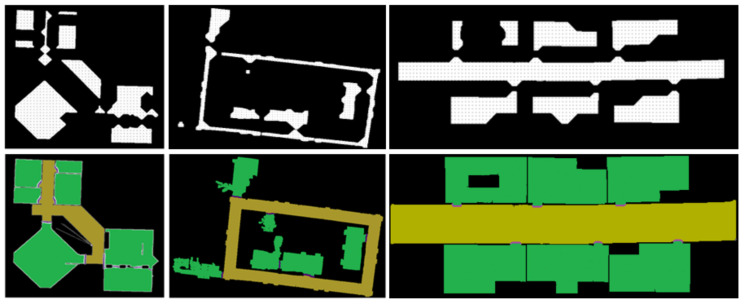
The experimental results of proposed method in three different maps.

The experimental results are shown in Figure 13, where the proposed algorithm shows amazing results on the three different 2D maps. The first row is the sampling diagram in the optimized sampling areas, the second row is the result of semantic segmented results. It can be seen that rooms, corridors and doorways are clearly labelled out in three different colors, while the number of sampling points of each map is greatly reduced. The specific results of the experiment are shown in Table 4. The average accuracy rate and average running time are 97.89% and 2.41 s, respectively, which is entirely acceptable for sweeping robots.

### 4.3. Comparison with Other Algorithms

**Figure 14:**
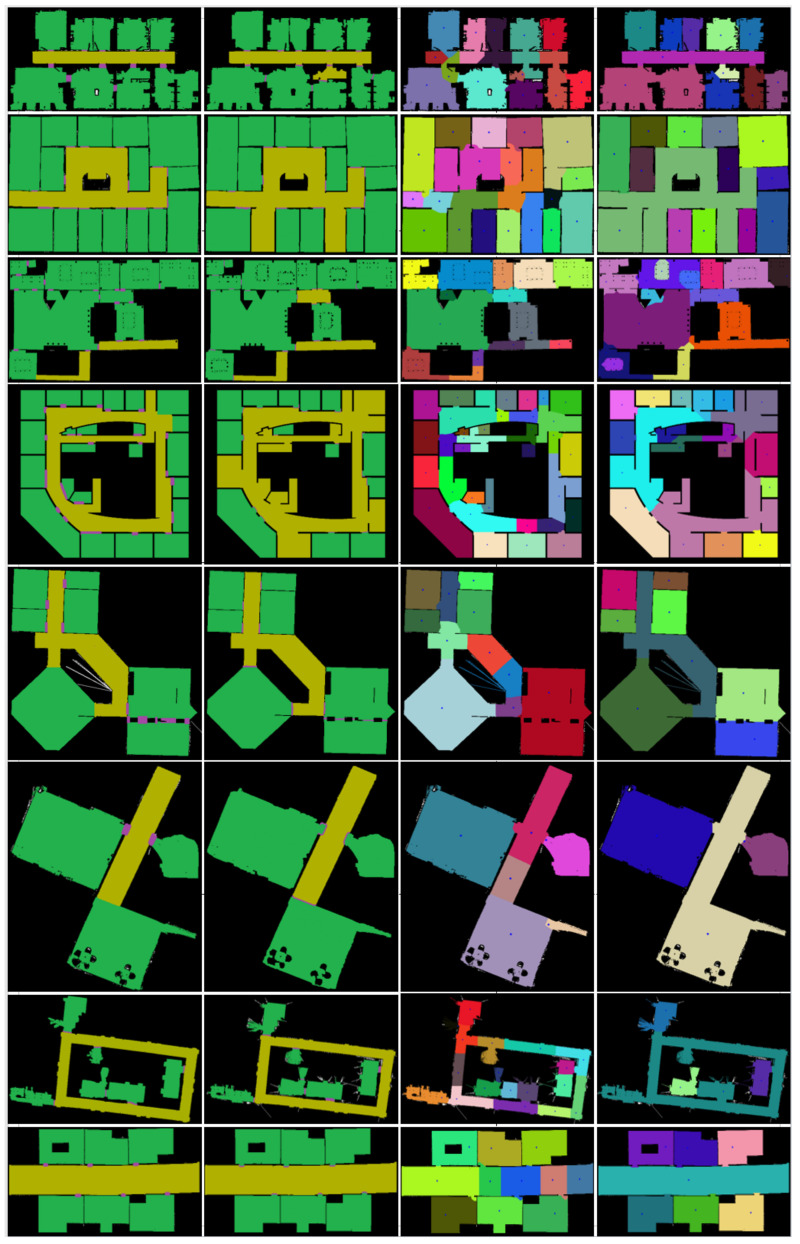
Exemplary segmentation results: the first column depicts the ground truth room segmentation from human labeling, the second column shows the proposed method’s segmentation, the third column yields the Voronoi graph-based segmentation, column 4 is the morphological-based segmentation.

In order to verify the performance of the proposed method, we used six maps of the public data set in [3] and two maps collected in our laboratory for comparison. The resolution of each map is 0.05 m/grid. The true area size of the maps ranges from 100 m^2^ to 1000 m^2^. Based on the above data, we used a unified hardware platform consisting of an Intel i7 8700 h CPU and an NVIDIA 1070ti GPU with 32 G RAM to compare the operation effect of the proposed algorithm with the Voronoi algorithm and the morphological segmentation method. The experimental results are shown in Figure 14, where the first column depicts the ground truth room segmentation from human labeling, the second column shows the proposed method’s segmentation, the third column depicts the Voronoi graph-based segmentation, and column 4 is the morphological-based segmentation. The average statistical results are shown in Table 5. The parameters in this table are explained as follows: *Recall*: the recall rate of the algorithm. *Precision*: the accuracy rate of the algorithm. *Average Runtime* [s]: the average running time of each map. *Segment area*: the average area of the obtained segments in m^2^. The evaluation method is expressed as follows:

(1) $$Accuracy=\frac{Correctly\;classified\;points}{All\;points}=\frac{TP+TN}{TP+FP+TN+FN}$$

(2) $$Precision=\frac{\sum_{i=0}^{2}TP_{i}}{\sum_{i=0}^{2}\left(TP_{i}+FP_{i}\right)}$$

(3) $$Recall=\frac{\sum_{i=0}^{2}TP_{i}}{\sum_{i=0}^{2}\left(TP_{i}+FN_{i}\right)}$$

where *TP* is True Positives, *FP* is False Positives, *FN* is False Negatives and *TN* is True Negatives.

It can be seen that the recall rate of the proposed method is the highest, which shows that the method has the least number of missed detections in the same dataset mentioned above. At the same time, in terms of accuracy, the proposed method has achieved the best results. In addition, the maximum average segmentation area is obtained, which shows that the segmentation effect of our method is better than the other two methods. Moreover, compared with the other two segmentation methods, the proposed method can obtain semantic labels accurately, which is of great significance for further application. While the others cannot get the semantic labels.

## 5. Conclusions

In this work, a new approach to get the semantic labels of 2D lidar room maps by combining the distance transform watershed-based pre-segmentation and a skillfully designed fast and efficient neural network lidar information sampling classification is proposed. A lidar is simulated to collect the lidar detection line maps of each point in the labelled map, and then these line maps and the corresponding labels are used to train the designed neural network, in the training stage. In the testing stage, the new map is first pre-segmented into various simple cells with the distance transformation watershed method, then we classify the lidar detection line maps sampled from these optimized sampling areas with the trained neural network. The speed of the proposed LCNet is 3.37 times faster than ResNet-18 on a PC, so the speed is significantly improved. Moreover, the size of the model is significantly reduced, and it can be extended to run on low computing power Raspberry Pi devices. After using the optimized sampling areas, the algorithm does not need to classify each point, which first improves the efficiency of the algorithm, secondly, due to the optimized sampling and the “winner takes all” classification principle, which effectively filters out the noise points of misclassification and improves the accuracy of the algorithm for semantic annotation. Comparing with the Voronoi algorithm and the morphological segmentation method, the recall rate and the accuracy rate of the proposed method are the highest, In addition, the segmentation effect of our method is better than those of the other two methods. Moreover, the proposed method can obtain semantic labels. Comparing with the distance-transform based method, our method not only can obtain the semantic information of maps, but also still run efficiently. A prototype mobile robot was developed to verify the proposed method, the feasibility, validity and high efficiency were verified by a series of tests. The proposed method achieved higher scores in its recall and precision. Specifically, the proposed method achieved a mean recall of 0.965 and a mean precision of 0.943.

## Figures and Tables

**Table 1:** The difference of convolution block between LCNet block and ResNet-18 bolck.

	ResNet-18	LCNet
Convolutional block	3 × 3 conv 3 × 3 conv	1 × 1 conv 3 × 3 Octconv 1 × 1 conv

**Table 2:** The layers of proposed LCNet.

Layers	LCNet	Output Size
Input		48 × 48
Block 1	1 × 1 conv 3 × 3 Octconv 1 × 1 conv	48 × 48
Transition layer	2 × 2 average pooling	24 × 24
Block 2	1 × 1 conv 3 × 3 Octconv 1 × 1 conv	24 × 24
Transition layer	2 × 2 average pooling	12 × 12
Block 3	1 × 1 conv 3 × 3 Octconv 1 × 1 conv	12 × 12
Transition layer	2 × 2 average pooling	6 × 6
Block 4	1 × 1 conv 3 × 3 Octconv 1 × 1 conv	6 × 6
Transition layer	2 × 2 average pooling	3 × 3
FC layer	256D FC layer	1 × 1
FC layer	512D FC layer	1 × 1
Classification layer	512D FC layer, softmax	1 × 1

**Table 3:** The running time of proposed LCNet and ResNet-18.

Network	Model Size	Sample Number of Test Set	Running Time on PC	Running Time on Raspberry PI
LCNet	3.4 M	8190	2.47 s	18.08 s
ResNet-18	44.7 M	8190	8.32 s	-

**Table 4:** The specific experimental results of the proposed method using in three different maps.

	Correctly Classfied Points/All Sampling Points	Accuracy Rate	Running Time
Map 1	578/589	98.13%	1.77 s
Map 2	662/682	97.06%	2.41 s
Map 3	774/786	98.47%	3.06 s

**Table 5:** The average general properties (± standard deviation) of the segmentation methods over 8 maps.

	Proposed Method	Voronoi	Morphological
Recall	96.5% ± 1.6%	93.5% ± 1.4%	94.7% ± 2.8%
Precision	94.3% ± 3.9%	86.6% ± 8.7%	91.3% ± 5.7%
Average Runtime (s)	2.83 ± 1.21	2.07 ± 1.06	1.25 ± 0.42
Segment area (m^2^)	58.2 ± 10.4	42.6 ± 8.5	39.1 ± 18.6
Segmented labels	Yes	No	No

## Data Availability

Not applicable.
